# Lipid metabolites of the phospholipase A_2_ pathway and inflammatory cytokines are associated with brain volume in paediatric cerebral malaria

**DOI:** 10.1186/s12936-015-1036-1

**Published:** 2015-12-21

**Authors:** Vasiliki Pappa, Karl Seydel, Sanchit Gupta, Catherine M. Feintuch, Michael J. Potchen, Samuel Kampondeni, Adam Goldman-Yassen, Mike Veenstra, Lillie Lopez, Ryung S. Kim, Joan W. Berman, Terrie Taylor, Johanna P. Daily

**Affiliations:** Department of Microbiology and Immunology, Albert Einstein College of Medicine, Bronx, NY 10461 USA; Blantyre Malaria Project, University of Malawi College of Medicine, Blantyre, Malawi; Department of Osteopathic Medical Specialties, College of Osteopathic Medicine, Michigan State University, East Lansing, MI 48824 USA; Department of Medicine (Infectious Diseases), Albert Einstein College of Medicine, Bronx, NY 10461 USA; Department of Radiology, University of Rochester, Rochester, NY 14642 USA; Lusaka Apex Medical University, Medical Radiation Sciences, Lusaka, Zambia; Department of Pathology, Albert Einstein College of Medicine, Bronx, NY 10461 USA; Department of Epidemiology and Population Health, Albert Einstein College of Medicine, Bronx, NY 10461 USA

**Keywords:** *Plasmodium falciparum*, Cerebral malaria, Brain swelling, Inflammation, Phospholipase A_2_, Plasma small molecules, Cytokines

## Abstract

**Background:**

Cerebral malaria (CM) remains a significant cause of morbidity and mortality in children in sub-Saharan Africa. CM mortality has been associated with increased brain volume, seen on neuroimaging studies.

**Methods:**

To examine the potential role of blood metabolites and inflammatory mediators in increased brain volume in Malawian children with CM, an association study was performed between plasma metabolites, cytokine levels and phospholipase A_2_ (PLA_2_) activity with brain volume.

**Results:**

The metabolomics analysis demonstrated arachidonic acid and other lysophospholipids to be positively associated with brain swelling. These lipids are products of the PLA_2_ enzyme and an association of plasma PLA_2_ enzymatic activity with brain swelling was confirmed. TNFα, which can upregulate PLA_2_ activity, was associated with brain volume. In addition, CCL2 and IL-8 were also associated with brain volume. Some of these cytokines can alter endothelial cell tight junction proteins and increase blood brain barrier permeability.

**Conclusions:**

Taken together, paediatric CM brain volume was associated with products of the PLA_2_ pathway and inflammatory cytokines. Their role in causality is unknown. These molecules will need to undergo testing in vitro and in animal models to understand their role in processes of increased brain volume. These observations provide novel data on host physiology associated with paediatric CM brain swelling, and may both inform pathogenesis models and suggest adjunct therapies that could improve the morbidity and mortality associated with paediatric CM.

**Electronic supplementary material:**

The online version of this article (doi:10.1186/s12936-015-1036-1) contains supplementary material, which is available to authorized users.

## Background

Infection with *Plasmodium falciparum* remains prevalent in many areas of the world and is associated with severe disease and mortality, particularly in children living in sub-Saharan Africa [1]. Cerebral malaria (CM) is a severe disease syndrome with mortality rates ranging from 15 to 25 % in research settings [2]. In addition, almost a third of paediatric CM survivors develop long-term neurological complications [2, 3]. Severe brain swelling seen on neuroimaging has been reported in paediatric CM. CM associated brain swelling is associated with poor outcomes in Kenyan children and is a significant predictor of mortality in Malawian children [4–6]. Recently, CM associated brain swelling determined by magnetic resonance imaging (MRI) found that swelling in survivors was readily reversible and that mortality was not associated with peripheral parasitaemia [5]. These observations have provided new insights into CM related morbidity and mortality and now can potentially inform development of adjuvant therapies to reverse or prevent brain swelling.

The mechanism of CM brain swelling is unknown and likely involves several factors including parasite mediated venous obstruction, increased permeability of the blood brain barrier (BBB), cytotoxic oedema or increased blood flow volume [5, 6]. Prior evidence of alterations in BBB permeability includes the observation of fibrinogen leakage into the brain [7]. Moreover, a reduction in endothelial cell tight junction proteins, which maintain the integrity of the BBB, has been reported, providing further support for increases in BBB permeability during CM [8–10]. A variety of systemic factors can lead to transient increase of BBB permeability in other diseases. These include the metabolic derangements associated with diabetic ketoacidosis and elevated concentrations of oxidized phospholipids [11, 12]. Systemic metabolic abnormalities are common in CM, which is often associated with a hyperlactataemia, hypoglycaemia and evidence of marked inflammation [13–16]. Therefore, metabolites measured in a cohort of Malawian children with CM were correlated to brain volume, to examine their role as potential mediators of brain swelling.

Arachidonic acid, other phospholipase A_2_ (PLA_2_) lipid metabolites and plasma PLA_2_ enzymatic activity were associated with brain swelling. Expression of the PLA_2_ enzyme is upregulated via the nuclear factor-kappa B (NFκB) pathway, which in turn is regulated by pro-inflammatory cytokines, such as tumour necrosis factor alpha (TNFα) [17]. An association of TNFα and other cytokines with brain swelling was found, suggesting that brain swelling is associated with a high inflammatory state. These data provide new biochemical insights into mechanisms of brain swelling in paediatric CM. Further experiments are needed to determine if these associated molecules induce increased brain swelling in the setting of CM.

## Methods

### Study population

To identify small molecules associated with brain swelling in paediatric CM, correlations were sought, between brain volume and both host factors and plasma metabolites in Malawian children enrolled in an ongoing study of malaria pathogenesis in the Blantyre Malaria Project (BMP) during the 2009, 2011 and 2013 transmission seasons. The BMP study enrolls children with clinically defined CM [2], who are between 6 months and 12 years of age. This analysis was restricted to children who were HIV negative, had negative blood and CSF bacterial cultures and evidence of malaria retinopathy [18, 19]. The study was restricted to patients with retinopathy positive CM, as the presence of retinal abnormalities increases the specificity of the clinical diagnosis of CM [18]. Plasma collected from the study subjects on admission, was stored at −80 °C and shipped to Albert Einstein College of Medicine in a liquid nitrogen dry shipper for subsequent metabolic and cytokine profiling. Plasma histidine rich protein 2 (HRP2), a parasite protein that represents total body parasite burden [20], was measured using ELISA, as previously described [21]. Clinical and laboratory data were extracted from the study database. Informed consent was obtained from the parent or guardian before enrollment into the BMP. This study was approved by the Albert Einstein College of Medicine Institutional Review Board (IRB), Michigan State University IRB, the University of Rochester IRB, and The University of Malawi College of Medicine Research and Ethics Committee.

### Neuroimaging

Neuroimaging was used to assess brain swelling with a 0.35T Signa Ovation Excite MRI scanner (GE Healthcare, Milwaukee, Wisconsin). The scans were read independently by two radiologists as previously reported [5, 6, 22]. Patient brain volume score was assigned based on a consensus interpretation of both radiologists. Brain volume was measured using a 1–8 scoring system, 1 and 2 indicating atrophy, 3-normal brain volume, 4-slightly increased brain volume, 5-mildly increased brain volume, 6-moderately increased brain volume, defined as loss of some sulcal markings, 7-moderately/severely increased brain volume with diffuse sulcal and cisternal effacement universally evident but without herniation present, and 8-severely increased brain volume with the findings of 7 and with evidence of herniation.

### Plasma cytokine quantification

Plasma cytokine data were available from a prior study (Feintuch, C.M. personal communication) for patients from 2009 and 2011. Cytokine levels were assessed by Luminex using the Human Cytokine Panel according to manufacturer’s instructions (Millipore) and read on a Magpix Multiplex Reader (Luminex).

### Metabolomics

Global non-targeted MS metabolomics analysis was performed at Metabolon, Inc. from 100 μL of plasma [23]. This method uses ultra-high performance liquid chromatography/tandem mass spectrometry in both positive and negative ion modes along with gas chromatography/mass spectrometry to maximize compound detection and accuracy. Metabolites were identified by comparing the spectral signatures of the plasma samples to a reference library using software developed at Metabolon [24].

### Plasma PLA_2_ activity fluorescence assay

To measure plasma PLA_2_ activity, a PLA_2_ fluorescence assay was performed as previously described with minor modifications [25]. Briefly, the liposomes were prepared with 2 mg of PG (l-α-Phosphatidyl-DL-glycerol sodium, Sigma) mixed with 14 μg of Red/Green BODIPY^®^ PC-A_2_ (1-*O*-(6-BODIPY^®^ 558/568-aminohexyl)-2-BODIPY^®^ FL C_5_-*sn*-glycero-3-phosphocholine, Life Technologies) in 1 ml of chloroform, which was subsequently evaporated under argon. The liposomes were added in 1 ml of (250 mM sucrose, 50 mM Tris–HCl pH 7.5 and 0.02 % sodium azide) and the mixture was vortexed for 20 min and sonicated 6 times for 30 s with 1 min intervals on ice. The liposomes were then aliquoted in smaller quantities, stored in −20 °C and used within 30 days of preparation. For each sample 3 μl of patient plasma were mixed with 97 μl of PLA_2_ assay buffer (10 mM Tris–HCl, pH 7.5 and 10 mM CaCl_2_) into a black assay plate (Corning). Subsequently, 1 μl of fluorescent phospholipid substrate was mixed with 99 μl PLA_2_ assay buffer, sonicated for 10 s and added to the assay plate for a final volume of 200 μl. The plate was shaken for 15 s, incubated at 37 °C for 30 min and fluorescence was recorded at 470 nm excitation and 515 nm emission (BioTek). Samples were run in triplicate and the mean values of the relative fluorescence units (RFUs) at 30 min are reported.

### Statistical analysis

To determine whether age, vital signs, HRP2, peripheral parasitaemia, plasma cytokines, complete blood count on admission, or coma resolution time correlated with brain volume, Spearman’s correlations were used. Two sided p-values <0.05 were considered statistically significant.

For the metabolomics analysis, ion counts were generated for each metabolite. A maximum likelihood method was used to impute left-truncated abundance values for each metabolite [26, 27]. First the mean and variance of the log transformed abundance values was estimated for each metabolite. Then the expectation of the left-truncated normal distribution of each metabolite was used to impute censored values. Spearman’s correlations were then performed between each metabolite or clinical factor and brain swelling scores.

The mean plasma PLA_2_ RFU values at 30 min of incubation were log10 transformed. Spearman’s correlations on the plasma PLA_2_ activity were then carried out and brain volume scores with p < 0.05 were considered statistically significant. The correlations were performed using Graph Pad Prism 6.03 (GraphPad Software, San Diego, CA, USA).

## Results

### Study population

Fifty-three Malawian children with retinopathy positive CM, who were enrolled in the BMP cohort during the transmission seasons 2009, 2011, and 2013, and had available plasma and neuro-imagining data were studied. Seven (13 %) children had a volume score of 3; eight (15 %) children had a volume score of 4; eleven (21 %) children had a volume score of 5; thirteen (25 %) children had a volume score of 6; and fourteen (26 %) children had a volume score of 7. The cohort had a median age of 52 months, haematocrit of 21.2 %, coma resolution time of 48 h, and 17 % mortality (Table 1). There were no statistically significant correlations between age, coma resolution time, vital signs, blood counts, peripheral parasitaemia or HRP2 concentration, measured on admission with brain swelling (Table 1) [5].

**Table 1:** Clinical and laboratory data of admission in 53 children with retinopathy positive CM and correlation with brain volume

Characteristic	Median (IQR)	P-value	Spearman’s r
Demographics			
Age (months)	52 (31.5–73)	0.88	−0.02
Female sex, no (%)	28 (53)		NA
Clinical findings			
Temperature (°C)	38.5 (37.6–39.4)	0.70	0.05
Blood pressure (mmHg)	93 (87–101)	0.14	−0.21
Heart rate (beats/min)	148 (129.5–163)	0.58	0.08
Respiratory rate (breaths/min)	42 (37–52)	0.67	0.06
Laboratory findings			
Parasitaemia (parasites × 10^3^/μl)	69.5 (22.0–338.8)	0.62	0.07
HRP2 (ng/ml)	7208 (2535–9541)	0.30	−0.15
Total WBC (×10^3^/μl)	7.9 (6.0–11.0)	0.28	0.15
Platelets (×10^3^/μl)	61.5 (30.8–100.5)	0.67	−0.08
Hct (%)	21.2 (17.9–26.4)	0.41	0.12
Clinical outcome			
Coma resolution time (h)	48 (30–82)	0.42	0.13
Death, no. (%)	9 (17)		NA

Description of patient characteristics and correlation with brain oedema scores. P-values and Spearman’s r are shown for continuous variables. IQR: interquartile range

### Metabolite correlation with brain volume

To identify plasma small molecules associated with CM brain swelling, a metabolomics analysis was carried out on 30 randomly selected plasma samples from the total study population enrolled in 2011 and 2013. There were no significant differences in the characteristics reported in Table 1 between the metabolomics sub-cohort and the total study population (Additional file 1: Table S1).

Brain volume positively correlated to 17 out of the 432 detected molecules (Spearman’s correlation, p value <0.05) (Table 2). A wide range of molecules was identified to be associated with brain volume, including lipids. Many of the significant lipid metabolites are PLA_2_ metabolites. PLA_2_ is an enzyme that hydrolyzes phospholipids at the sn-2 position, to liberate lysophospholipids and free fatty acids, including arachidonic acid [28]. Brain swelling correlated with the lysophospholipids, 1-eicosatrienoylglycerophosphoethanolamine and 1-oleoylglycerophosphoethanolamine. The fatty acids arachidonic acid and pentadecanoic acid, as well as 5-hydroxyhexanoate, a fatty acid metabolite, were also significantly associated with brain swelling.

**Table 2:** Plasma metabolites associated with brain volume in Malawian children with retinopathy positive CM

Metabolite	P-value	Spearman’s r
Lipids associated with the PLA_2_ pathway		
Pentadecanoate (15:0)	0.03	0.39
1-Eicosatrienoylglycerophosphoethanolamine*	0.04	0.38
Arachidonate (20:4n6)	0.04	0.38
1-Oleoylglycerophosphoethanolamine	0.05	0.37
Other lipids		
Butyrylcarnitine	0.01	0.46
2-Linoleoylglycerol (2-monolinolein)	0.02	0.42
Octanoylcarnitine	0.03	0.41
5-Hydroxyhexanoate	0.04	0.37
Other metabolites		
Phenylacetate	0.00	0.52
1,3,7-trimethylurate	0.00	0.52
Alpha-ketoglutarate	0.01	0.46
Mannitol	0.01	0.45
3-hydroxy-2-ethylpropionate	0.02	0.43
Paraxanthine	0.02	0.42
Thymol sulfate	0.04	0.38
l-urobilin	0.04	0.37
Sucrose	0.04	0.37

Analysis carried out on from 30 samples collected in 2011 and 2013 are shown (p-value <0.05). Asterisk represents a metabolite that was identified based on its chromatographic and mass spectra rather than a purified standard

l-urobilin, a metabolite of the hemoglobin catabolism pathway, was highly correlated with brain volume. Among other molecules, the carbohydrate, mannitol was also associated with brain swelling (Table 2).

### Plasma sPLA_2_ activity and CM brain swelling

**Fig. 1:**
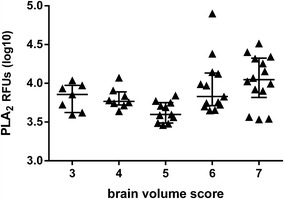
Plasma PLA_2_ activity correlates to brain volume in Malawian children with retinopathy positive CM; Plasma PLA_2_ activity correlates to brain volume. (Spearman’s correlation r = 0.31, p-value = 0.02) Medians and interquartile range are depicted. *RFU* relative fluorescence units

Since PLA_2_ metabolites correlated with brain swelling, the PLA_2_ enzymatic activity itself was examined for correlations with brain swelling. To determine PLA_2_ activity, a fluorescence PLA_2_ enzymatic assay was carried out on the same BMP study enrollment plasma samples from all 53 patient samples. A significant correlation of increasing PLA_2_ activity and brain volume was observed (Spearman’s correlation r = 0.31, *p* value = 0.02) (Fig. 1).

### Cytokine activity and CM brain swelling

**Fig. 2:**
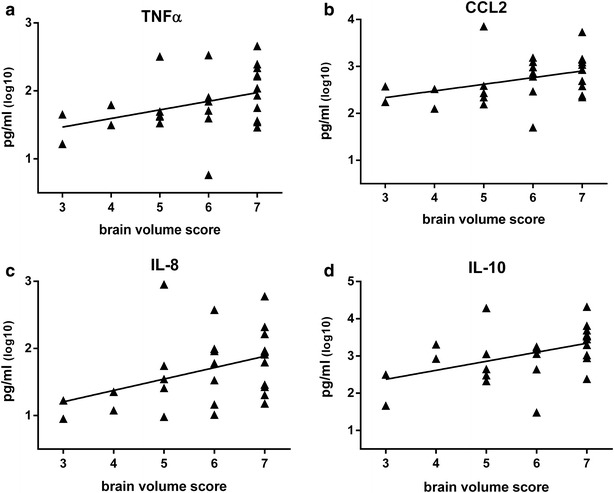
Cytokines correlate to brain volume in Malawian children with retinopathy positive CM; Multiple cytokines significantly correlate with brain volume. **a** TNFα levels (Spearman’s correlation r, 0.38, p-value = 0.05). **b** CCL2 levels (Spearman’s correlation r, 0.44, p-value = 0.02). **c** IL-8 levels (Spearman’s correlation r, 0.43, p-value = 0.03). **d** IL-10 levels (Spearman’s correlation r: 0.50, p-value = 0.01). Cytokines were measured by Luminex in duplicate and the mean value is displayed

The PLA_2_ pathway and its downstream lipid products, such as arachidonic acid mediate inflammatory responses [29]. PLA_2_ can be upregulated by TNFα and thus we examined TNFα and other inflammation related cytokines for their association with CM brain swelling. Cytokine data were available for 27 samples from the total cohort. For this subset of patients, clinical and laboratory data were similar to the total study population (Additional file 1: Table S1). Statistically significant correlations were observed, between brain volume, and TNFα (Spearman’s correlation r = 0.38, p-value = 0.05), CCL2 (Spearman’s correlation r = 0.44, p-value = 0.02), IL-8 (Spearman’s correlation r = 0.43, p-value = 0.03) and IL-10 (Spearman’s correlation r = 0.50, p-value = 0.01) (Fig. 2).

## Discussion

To identify potential mediators of brain swelling associated with paediatric CM, plasma metabolite levels of Malawian children with retinopathy positive CM were correlated to brain volume. Associations were identified between PLA_2_ metabolites, including arachidonic acid, with brain volume. Plasma PLA_2_ enzymatic activity also correlated to brain volume, suggesting an upregulation of the PLA_2_ pathway in children with high brain swelling. TNFα can upregulate PLA_2_ and this study reports that, in conjunction with other inflammatory cytokines, TNFα is correlated with brain volume. l-urobilin, a haem degradation product and mannitol were also associated with brain volume. Collectively, these results suggest a higher inflammatory state in children with increased brain volume. Further studies on how these molecules may be involved in BBB disruption, and why some children sustain a higher inflammatory state are now warranted.

Brain swelling has recently been shown to be the strongest predictor of mortality in paediatric CM, with an adjusted odds ratio of 7.5 (95 % CI 2.1–26.9) for severe brain swelling among patients who died compared to those who survived [5]. Brain swelling induces increased intracranial pressure that may lead to brain-stem compromise and respiratory arrest. The mechanism of increased brain volume is unknown. Multiple mechanisms of increased brain volume may exist in clinical conditions such as CM [30], including processes that impact BBB function [5, 7, 10]. This study set out to identify circulating biochemical mediators that may contribute to BBB dysfunction. [7, 10].

Positive correlations between brain volume and several lipids that are PLA_2_ metabolites were identified. The fatty acids released by PLA_2_, such as arachidonic acid, can be sources of energy, signaling, and of relevance to this study, potent mediators of inflammation. Specifically, arachidonic acid, the precursor of the eicosanoid pathway, can increase the permeability of human brain microvasculature endothelial cell monolayers via prostaglandin E_2_ activation of EP_3_ and EP_4_ receptors [31]. Lysophospholipids are also generated by PLA_2_ and are important in cell signaling and membrane biology. PLA_2_ enzymatic activity has been associated with neurological and inflammatory conditions and inhibitors of the PLA_2_ enzyme are being studied to reduce pathologic inflammation [32–36]. Fatty acids and lysophospholipids can be generated by enzymes other than PLA_2_, therefore it was confirmed in this study that plasma PLA_2_ activity was also correlated with brain swelling [37, 38]. These data extend a prior observation where high PLA_2_ plasma activity was associated with severe malaria and death in Malawian children [39]. Why some children have higher PLA_2_ enzyme activity is unknown. PLA_2_ activity is tightly regulated by host responses including TNFα and reactive oxygen species (ROS), both of which can be elevated during severe malaria [15, 40, 41]. Thus the PLA_2_ pathway and its metabolites may be acting directly on brain microvasculature endothelial cells or indirectly through their effects on cell signaling or energy metabolism.

l-urobilin, a degradation product of haem also correlated with brain volume. Malaria induces erythrocyte lysis and subsequent release of haemoglobin into the circulation. This results in increase of bloodstream ROS levels, which can alter BBB permeability [41–44]. Lysed erythrocytes can increase BBB permeability and result in brain oedema, typically occurring twenty-four hours after haemolysis [45–47]. Direct measurement of ROS and other haemoglobin metabolites are needed to explore the role of erythrocyte lysis in CM associated brain swelling.

Plasma mannitol was also found to have a positive correlation with brain swelling. Exogenous mannitol has been associated with increased brain oedema or brain weight in animal models of vasogenic oedema and brain infarct [50, 51]. Mannitol is used therapeutically for the treatment of increased intracranial pressure following brain injury [48, 49]. Mannitol adjunctive therapy has been studied in CM and was found to either have no significant effect on clinical outcomes in paediatric CM in Uganda [52] or to potentially have adverse outcomes by prolonging coma resolution time in adults with CM [53]. Further studies to investigate the role of endogenous and exogenous mannitol in CM paediatric brain swelling are necessary.

Inflammatory cytokines were also associated with brain volume and these may directly alter BBB permeability. CCL2 and IL-8 have been previously associated with alterations in tight junction proteins including occludin, ZO-1 and claudin-5 resulting in increased brain endothelial cell permeability [54, 55]. In addition, TNFα has been shown to increase retinal endothelial cell permeability through protein kinase C zeta (PKCζ) and the NFκB pathway by reducing the expression and distribution of claudin-5 and ZO-1 [56]. Interestingly, inhibition of TNFα by soluble TNF p55 receptor attenuates status epilepticus-induced oedema in a rat model, which could be relevant in CM, as seizures are highly prevalent [3, 57]. Furthermore, TNFα can regulate the transcription of PLA_2_ proteins via the NFκB pathway, providing a link between inflammatory cytokines and the PLA_2_ pathway [17]. The mechanism, where inflammatory cytokines, increased PLA_2_ activity and its lipid metabolites converge to disrupt the BBB, in the setting of parasites sequestration to brain endothelial cells in CM, requires further investigation. If the PLA_2_ pathway is found to play a direct role in BBB dysfunction, PLA_2_ inhibitors could be evaluated as potential adjunctive therapy.

The higher inflammation and brain volume do not correlate with peripheral parasitaemia or total parasite body load represented by parasite HRP2 levels [58]. The variability in inflammatory responses during infection could be attributed to differences in disease duration, host or parasite genetic polymorphism, prior malaria exposures, uric acid levels or other host or parasite factors [59–65]. Further studies examining how host and/or parasite parameters are mediating inflammation and upregulation of the PLA_2_ pathway in the setting of increased brain volume could inform therapeutic intervention.

A limitation to the design of this study is that the plasma metabolome was examined after onset of brain swelling. For this reason, it is difficult to know if the metabolic profile is a cause of BBB dysfunction or the result of changes secondary to increased brain volume. A longitudinal study design with repeated metabolic sampling over time in correlation to brain swelling status would provide a more powerful approach to identify potential mediators of BBB permeability. Additionally, there were no metabolomics data from patients with brain volume scores 1, 2 and 8 due to the low prevalence of those brain volume scores. To develop biomarkers of brain swelling for regions without MRI, further plasma analyses that include all of the brain volume groups are warranted. Given the variances observed, larger sample sizes would permit corrections for multiple comparisons.

## Conclusions

This study shows associations between brain volume and plasma PLA_2_ activity, metabolites of the PLA_2_ pathway, inflammatory cytokines, and other molecules in paediatric CM patients. The role of these small molecules and cytokines as disease mediators or drug target candidates can now be further investigated.

## Additional file

10.1186/s12936-015-1036-1 Clinical and laboratory data of admission in children with retinopathy positive CM and comparison between sub-cohorts and total study population.

## Abbreviations

CM: cerebral malaria
PLA_2_: phospholipase A_2_
TNF: tumour necrosis factor
CCL2: chemokine (C–C motif) ligand 2
IL-8: interleukin 8
MRI: magnetic resonance imaging
BBB: blood brain barrier
BMP: Blantyre Malaria Project
HIV: human immunodeficiency virus
CSF: cerebrospinal fluid
iRBC: infected red blood cell
IRB: Institutional Review Board
MS: mass spectrometry
PG: l-α-phosphatidyl-DL-glycerol sodium
Red/Green BODIPY^®^ PC-A_2_: 1-*O*-(6-BODIPY^®^ 558/568-aminohexyl)-2-BODIPY^®^ FL C_5_-*sn*-glycero-3- phosphocholine
RFU: relative fluorescence unit
IL-10: interleukin 10
ROS: reactive oxygen species
ZO-1: zonula occludens-1
PKCζ: protein kinase C zeta
NFκB: nuclear factor-kappaB
HRP2: histidine rich protein 2
