# Supplementary material for: Lipid metabolites of the phospholipase A2 pathway and inflammatory cytokines are associated with brain volume in paediatric cerebral malaria
Source: Malar J. 2015 Dec 21;14:513. doi: 10.1186/s12936-015-1036-1 (PMC4687364; doi:10.1186/s12936-015-1036-1)
Supplement: Supplementary file 1 — 10.1186/s12936-015-1036-1 Clinical and laboratory data of admission in children with retinopathy positive CM and comparison between sub-cohorts and total study population. [file 12936_2015_1036_MOESM1_ESM.docx]

**Supplemental Table. Clinical and laboratory data of admission in children with retinopathy positive CM and comparison between sub-cohorts and total study population.**

| Characteristic | **Study population** | **Metabolomics cohort** | | **Cytokine cohort** | |
| --- | --- | --- | --- | --- | --- |
|  | **Median (IQR)**  n = 53 | **Median (IQR)** n = 30 | **P-value** | **Median (IQR)** n = 27 | **P-value** |
| Demographics |  |  |  |  |  |
| Age (months) | 52 (31.5-73) | 54 (33-72.5) | 0.92 | 53 (34-67) | 0.881 |
| Female sex, No (%)* | 28 (53) | 15 (50) | 0.82 | 16 (59) | 0.64 |
| **Clinical Findings** |  |  |  |  |  |
| Temperature (°C) | 38.5 (37.6-39.4) | 38.1 (37.4-39.1) | 0.44 | 38.9 (37.9-39.5) | 0.399 |
| Blood pressure (mmHg) | 93 (87-101) | 94 (87.5-102.5) | 0.88 | 93 (89.5-100.5) | 0.902 |
| Heart Rate (beats/min) | 148 (129.5-163) | 149 (128.8-164.5) | 0.88 | 147 (130-161) | 0.994 |
| Respiratory Rate (breaths/min) | 42 (37-52) | 44 (39.5-54) | 0.68 | 40 (36-48) | 0.324 |
| **Laboratory Findings** |  |  |  |  |  |
| Parasitaemia (parasites/μl) | 69,480 (21,960-338,790) | 58,430 (8,830-197,835) | 0.63 | 71,280 (24,300-331,740) | 0.97 |
| HRP2 (ng/ml) | 7,208 (2,535-9,541) | 6,328 (1,283-8,373) | 0.44 | 8,292 (2,882-9,916) | 0.477 |
| Total WBC (x10^3^/μl) | 7.9 (6.0-11.0) | 7.9 (5.8-11.0) | 0.99 | 7.9 (6.7-9.6) | 0.953 |
| Platelets (x10^3^/μl) | 61.5 (30.8-100.5) | 61.5 (30.3-96.3) | 0.81 | 36 (30.0-146) | 0.798 |
| Hct (%) | 21.2 (17.9-26.4) | 21.0 (17.3-27.5) | 0.89 | 20.8 (17.9-24.1) | 0.553 |
| **Clinical Outcome** |  |  |  |  |  |
| Coma resolution time (hrs) | 48 (30-82) | 58 (35-92) | 0.39 | 43 (28-63.5) | 0.476 |
| Death, No. (%)* | 9 (17) | 5 (16.7) | 1 | 4 (14.8) | 1 |

Comparison of patient characteristics between study population and cohorts. P-values for Mann–Whitney test comparison between total study population and each cohort are shown. Asterisk denotes variables for which Fisher's exact test was performed. P-values < 0.05 were considered statistically significant. IQR: interquartile range
